# Exploring Dual-to-Single-Encoder Transitions for Real-Time RGB-T Semantic Segmentation

**DOI:** 10.3390/s26206602

**Published:** 2026-10-18

**Authors:** Junghwan Kim, Minkyu Jung, Byungin Moon

**Affiliations:** 1 Graduate School of Electronic and Electrical Engineering, Kyungpook National University, Daegu 41566, Republic of Korea; 2 School of Electronics Engineering, Kyungpook National University, Daegu 41566, Republic of Korea

**Keywords:** RGB-T semantic segmentation, multimodal fusion, encoder transition, computational efficiency, real-time perception

## Abstract

RGB-T semantic segmentation improves scene understanding under adverse illumination by exploiting complementary visible and thermal cues. However, conventional intermediate fusion networks preserve two modality-specific encoder branches and repeatedly apply fusion modules throughout the feature hierarchy, increasing the parameter count, computational cost, and inference latency. To address this issue, we propose a dual-to-single-encoder transition strategy that retains modality-specific branches only up to a designated transition stage, applies an explicit attention-enhanced fusion (EAEF) operation at that stage, and processes the remaining encoder stages through a single shared branch. Stage-wise representational alignment is examined using linear centered kernel alignment between independently trained RGB-only and thermal-only encoders, providing a fusion-independent rationale for treating the transition stage as an architectural design variable. Transition stages s0–s4 are evaluated with ResNet-18, ResNet-50, and ResNet-152 backbones on MFNet and FMB. The selected EAEFNet-ResNet-50-s1 configuration reduces parameters and floating-point operations (FLOPs) by 62.6% and 28.5%, respectively, relative to the native EAEFNet-ResNet-50, while improving MFNet mean Intersection over Union (mIoU) by 2.7 percentage points and increasing throughput from 30.4 to 45.9 frames per second (FPS). It also achieves 60.5% mIoU on FMB. These results demonstrate that an early-to-middle encoder transition can preserve competitive segmentation accuracy while providing a favorable accuracy–efficiency trade-off for efficient RGB-T perception.

## 1. Introduction

Accurate and reliable perception of the surrounding driving environment is essential for the development of advanced driver-assistance systems (ADAS) and autonomous vehicle technologies [1,2,3,4]. Semantic segmentation has been widely studied as a fundamental perception task for intelligent driving systems because it assigns a semantic label to each image pixel and thereby enables pixel-level delineation of driving-relevant regions and objects, including roads, vehicles, pedestrians, and obstacles [5]. Because perception outputs in real-world driving scenarios are directly linked to downstream control and decision-making, such systems must satisfy stringent real-time processing requirements in addition to achieving high segmentation accuracy.

Conventional semantic segmentation research has largely focused on RGB images. Although RGB images provide rich color and texture information, their reliability can degrade substantially under adverse conditions such as nighttime scenes, low illumination, fog, abrupt illumination changes, glare, and reflections. This limitation indicates that machine-learning models trained on a single sensing modality may exhibit condition-induced bias toward specific visual environments. Accordingly, multimodal data fusion techniques that exploit sensors with different physical characteristics have attracted increasing attention as a promising way to mitigate such sensor-data bias and improve robustness in semantic segmentation [6,7]. In this context, thermal cameras capture infrared radiation emitted by objects and are less dependent on ambient illumination, allowing thermal images to provide complementary cues when objects are difficult to distinguish in RGB images. Such complementary sensing has direct practical value in autonomous platforms. Vehicle-mounted visible–thermal camera systems have been evaluated for pedestrian detection across daytime and nighttime traffic scenes [8], while RGB and long-wave infrared (LWIR) cameras have also been mounted onboard a mobile robot for human detection [9]. In search-and-rescue robotics, RGB, thermal, and multispectral cameras have further been deployed on a quadruped robot to evaluate victim detection in indoor and outdoor environments [10]. Accordingly, RGB-T semantic segmentation has been studied as a promising approach for improving scene understanding under variable illumination conditions [11].

Although existing networks have achieved high accuracy in RGB-T semantic segmentation, they still face important limitations when targeting resource-constrained deployment because of their encoder architecture [12]. An encoder that maintains modality-specific RGB and thermal branches is advantageous for learning modality-specific representations from RGB and thermal images [13], but it increases the number of parameters and floating-point operations (FLOPs) relative to an encoder that uses a single shared branch. In addition, multi-level fusion can improve accuracy by repeatedly performing cross-modal feature interaction across encoder stages; however, the computational cost and latency introduced by the fusion modules can accumulate. In particular, complex attention- or transformer-based modules [14,15] provide high representational capacity, but their nonlinear operations and memory access overhead can impose a considerable burden on real-time inference in practical deployments where computational efficiency is highly desired. Previous studies have primarily focused on designing more elaborate mechanisms for fusing RGB features and thermal features. However, comparatively limited attention has been paid to the branch topology itself: up to which encoder stage the modality-specific RGB and thermal branches should be preserved, and from which stage the network should use a single shared branch. This issue is particularly important when considering practical deployments, where segmentation accuracy, the number of parameters, computational cost, and inference throughput, typically measured in frames per second (FPS), should be considered jointly. Therefore, efficient architecture design requires consideration of both the fusion module and the transition stage.

In this paper, we focus on designing a lightweight and efficient RGB-T semantic segmentation architecture for computationally efficient processing. To this end, we propose a dual-to-single-encoder transition strategy for real-time RGB-T semantic segmentation. The proposed strategy extracts RGB and thermal features through modality-specific branches up to a designated transition stage, fuses the two feature streams once at that stage, and uses a single shared branch for the subsequent encoder stages. This design aims to retain the benefits of modality-specific representation learning while reducing parameters, FLOPs, and inference latency caused by maintaining modality-specific branches in later encoder stages and repeatedly applying multi-level fusion. The main contributions of this study are summarized as follows:

- We propose a dual-to-single-encoder transition strategy for RGB-T semantic segmentation, in which modality-specific RGB and thermal branches are maintained up to a designated transition stage, after which the two feature streams are fused and processed using a single shared branch.
- We provide a systematic transition-stage analysis from s0 to s4 across the ResNet-18, ResNet-50, and ResNet-152 backbones [16] on the MFNet [17] and FMB [18] urban RGB-T semantic segmentation datasets.
- We show that early-to-middle transition stages can reduce parameters and computational cost while maintaining competitive segmentation accuracy, thereby improving the accuracy–efficiency trade-off.
- We present an efficient design perspective for RGB-T semantic segmentation by treating the encoder transition stage as an architectural design variable rather than relying solely on increasingly complex fusion modules.

The remainder of this paper is organized as follows: Section 2 reviews related work on RGB-T semantic segmentation, modality fusion strategies, and the EAEFNet baseline. Section 3 presents the proposed network architecture, analyzes the transition stage, and establishes the representative network configuration. Section 4 describes the experimental setup and reports quantitative and qualitative comparisons on the MFNet and FMB datasets. Finally, Section 5 concludes the paper and discusses directions for future work.

## 2. Related Work

### 2.1. RGB-T Semantic Segmentation

Visible-spectrum (RGB) images deliver rich spatial textures and chromatic details, making them the cornerstone of the semantic segmentation task in intelligent transportation systems. However, their reliability drops drastically in adverse driving environments, such as nocturnal conditions, dense fog, or sudden illumination transitions. To avoid these modality-specific vulnerabilities, the integration of thermal infrared (T) sensors has emerged as a crucial paradigm. Thermal sensors capture emitted long-wave infrared radiation, making them highly resilient to ambient lighting variations and enabling reliable delineation of heat-emitting targets (e.g., pedestrians and operating vehicles) in low-light environments, although their effectiveness can still be influenced by specific environmental and sensing conditions.

Early works in this domain focused on constructing paired datasets to benchmark the multimodal task, notably the MFNet dataset and more recently the FMB dataset. Following these data-driven foundations, numerous deep-learning architectures have been proposed to effectively interpret cross-modal inputs. The evolution of these architectures has fundamentally revolved around minimizing the semantic gap between the structural characteristics of RGB data and the statistical radiation distributions of thermal data. Beyond automotive perception, the broader utility of advanced vision devices has been demonstrated in diverse domains, including the tribological monitoring of mechanical components [19]. Within the specific context of RGB-T semantic segmentation, recent methodologies have also advanced fusion efficiency by employing hierarchical feature enhancement and progressive weight-sharing mechanisms [20].

### 2.2. Modality Fusion Strategies

The methodology of combining heterogeneous features is broadly divided into early, late, and intermediate fusion based on the stage of multimodal integration [21].

Early fusion techniques concatenate the RGB and thermal inputs at the initial pixel level, passing a multi-channel tensor (e.g., four-channel R-G-B-T) into a standard single-stream backbone [22]. Although this paradigm possesses architectural simplicity and has a low computational cost, it typically delivers suboptimal segmentation boundaries. Because raw data streams are intermingled prior to learning modality-specific representations, the distinct physical properties of thermal and RGB sensors tend to suppress each other, leading to a loss of complementary cues. In contrast, late fusion integrates modality-specific information through high-level features or near the prediction stage [23]. This approach preserves modality-specific representation learning but provides limited opportunities for complementary cross-modal interaction at intermediate stages.

To balance modality-specific representation learning with cross-modal interaction, intermediate fusion has become the dominant approach in RGB-T semantic segmentation. This approach maintains dual-branch encoders to extract independent feature hierarchies from RGB and thermal paths simultaneously, utilizing dedicated fusion modules in multiple stages. Fusion mechanisms range from simple arithmetic operations, such as element-wise summation or multiplication [24], to attention-based and transformer-based architectures. For instance, attention-based fusion methods utilize channel and spatial attention mechanisms, such as variations in the convolutional block attention module (CBAM), to dynamically weight feature maps based on environmental contexts, effectively suppressing the unreliable RGB branch under nighttime or low-illumination conditions. More recently, transformer-based fusion frameworks have deployed cross-attention mechanisms to capture long-range dependencies and global contextual alignments between the two heterogeneous modalities, establishing robust cross-modal representations.

Despite their empirical success, these state-of-the-art intermediate fusion networks suffer from severe structural redundancy. Preserving dual symmetric branches throughout the entire depth of heavy backbones drastically escalates the number of parameters and FLOPs. Furthermore, the cascade of multi-level attention or transformer blocks introduces nonlinear operations and excessive memory access overhead, making them highly impractical for real-time inference in computationally limited environments.

While the aforementioned methods predominantly rely on symmetric dual-branch architectures, some prior works have explored asymmetric streams [25,26] or progressive multi-modal fusion [27] to reduce computational overhead. However, these approaches often require complex architectural redesigns or custom fusion operators. In contrast, our proposed topology is distinct in that it introduces a straightforward dual-to-single-encoder transition that can be integrated with existing explicit fusion mechanisms without altering the underlying fusion operator itself. This allows us to isolate and realize the efficiency gains derived purely from branch topology optimization.

### 2.3. EAEFNet

To achieve a more balanced trade-off between segmentation accuracy and computational efficiency, recent research has gravitated toward optimizing the fusion architecture itself. A representative baseline addressing this challenge is the Explicit Attention-Enhanced Fusion Network (EAEFNet) [28]. EAEFNet operates on a multi-level intermediate fusion framework, systematically designed to leverage explicit attention mechanisms to guide cross-modal interaction rather than relying on implicit feature blending. Structurally, EAEFNet maintains distinct encoder branches for RGB and thermal inputs, integrating explicit attention modules at multiple hierarchical stages. These modules calculate explicit correlation weights to dynamically align the heterogeneous feature maps, actively suppressing cross-modal noise and reinforcing complementary cues where the modal gap is most severe. By incorporating explicit attentional guidance, EAEFNet successfully captures the intricate dependencies between RGB and thermal features, demonstrating robust segmentation performance under complex driving scenarios. However, a critical bottleneck remains unaddressed within the EAEFNet architecture regarding its branch topology. Although EAEFNet introduces explicit attention to streamline cross-modal interaction efficiently, it still conforms to the conventional paradigm of maintaining separate, symmetric dual-branch encoder paths throughout all hierarchical stages (from the shallowest to the deepest spatial resolutions). While this prolonged dual-branch configuration ensures that modality-specific feature representations are well-preserved for the explicit attention modules, it inevitably increases the backbone parameter count and computational cost.

Under computational constraints, continuing to extract independent features in the later encoder stages offers diminishing returns in segmentation accuracy, while introducing severe latency and memory access overhead. This trade-off highlights that optimizing the fusion module alone is insufficient for extreme resource constraints. Consequently, this limitation motivates our investigation into a dual-to-single-encoder transition strategy, exploring exactly at which architectural stage the modality-specific branches can be unified into a single shared branch without compromising the benefits of explicit attention-guided feature representation.

## 3. Proposed Method

### 3.1. Architecture Overview

**Figure 1:**
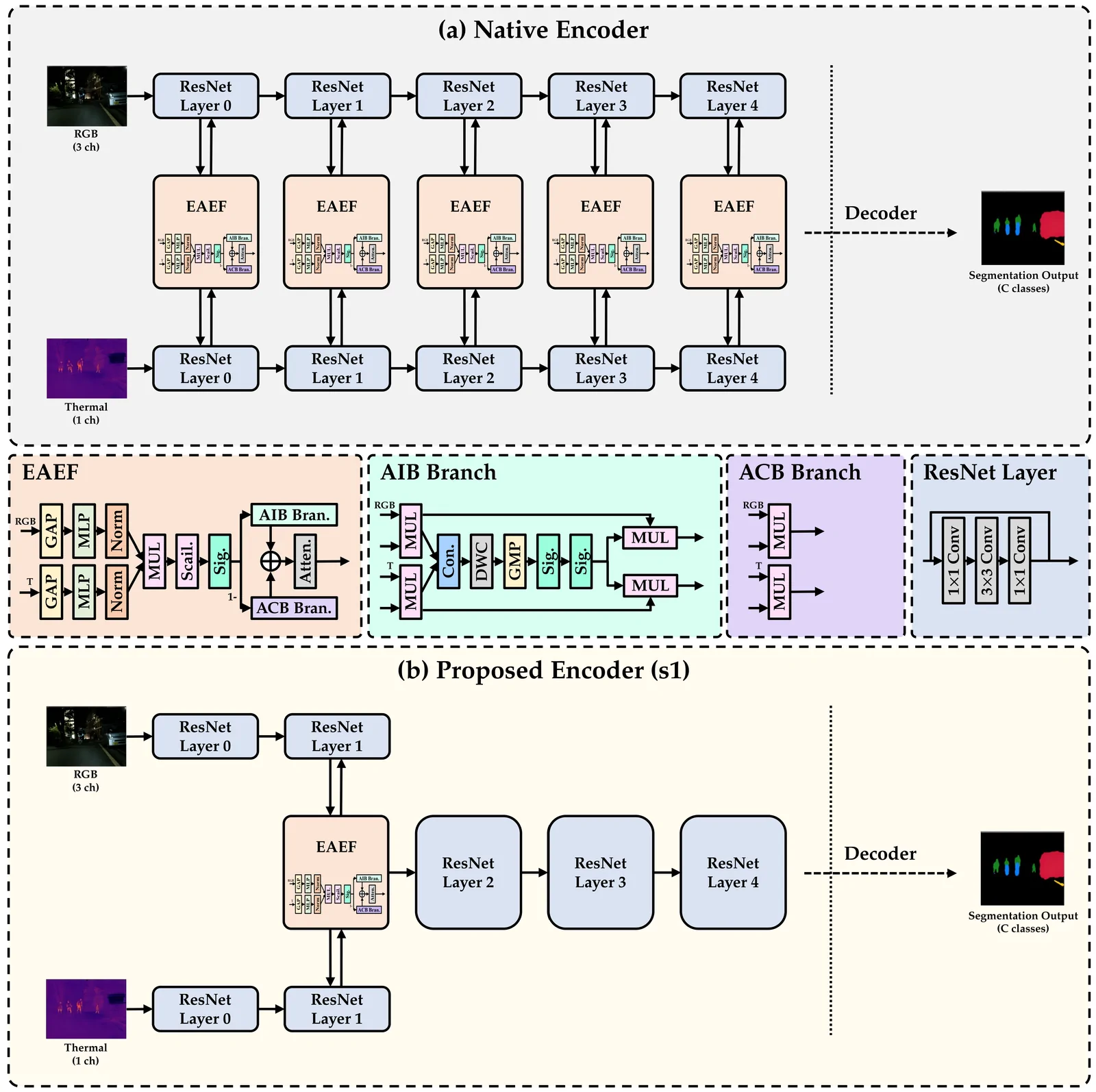
Overview of the proposed dual-to-single-encoder transition strategy. (**a**) Native EAEFNet encoder, where modality-specific RGB and thermal branches are maintained throughout the encoder and EAEF operations are applied across stages. The middle row illustrates the internal structures of the EAEF operation and the ResNet layer used in the encoder. (**b**) Proposed encoder with an example transition at s1. After the selected transition stage, the fused representation is routed through a single shared branch for the remaining encoder stages, while the decoder remains unchanged.

The proposed network takes paired RGB and thermal images as input and produces a semantic prediction map through an RGB-T encoder–decoder architecture. Figure 1 illustrates the difference between the native EAEFNet encoder and the proposed dual-to-single-encoder transition strategy. As shown in Figure 1a, the native topology maintains modality-specific RGB and thermal branches throughout the encoder and applies explicit attention-enhanced fusion (EAEF) operations across stages. This design explicitly preserves modality-specific representations, but it also keeps both encoder branches active until the deepest stage.

In contrast, Figure 1b shows the proposed topology. The modality-specific RGB and thermal branches are preserved only up to a selected transition stage. At this stage, the corresponding EAEF operation forms a cross-modal representation, which is then routed through a single shared branch for the remaining encoder stages. As a result, thermal-side encoder stages after the transition stage are excluded from the active forward path, while the multimodal cues obtained before and at the transition stage are propagated to the subsequent encoder stages.

Let the input RGB image and thermal image be denoted by $I^{R}$ and $I^{T}$, respectively. The encoder stages are indexed by k∈{0,1,2,3,4} and denoted as s0–s4. Here, s0 represents the stem-level feature stage, whereas s1–s4 correspond to the ResNet residual stages. Let $R_{k}$ and $T_{k}$ be the RGB and thermal feature maps at stage *k*, and let $F_{k}^{\mathrm{EAEF}}$ denote the EAEF operation at the same stage. The transition index is denoted by τ∈{0,1,2,3,4}.

At the selected transition stage τ, the RGB and thermal features are combined using the corresponding EAEF operation:

(1) $$\left({\hat{R}}_{\tau },{\hat{T}}_{\tau }\right)=F_{\tau }^{\mathrm{EAEF}}\left(R_{\tau },T_{\tau }\right),\;Z_{\tau }={\hat{R}}_{\tau }.$$

Here, $Z_{\tau }$ denotes the fused representation passed to the single shared branch. Although $Z_{\tau }$ is represented by the RGB-aligned output, it has already been updated through EAEF-based interaction with the thermal feature and therefore carries cross-modal information.

The remaining encoder stages are then executed once through the single shared branch:

(2) $$Z_{k}=E_{k}^{S}\left(Z_{k-1}\right),\;k=\tau +1,\ldots ,4,$$

where $E_{k}^{S}$ denotes the stage-wise operation in the single shared branch. To be architecturally explicit, this shared continuation path is physically instantiated using the remaining stages of the RGB-side encoder. This design choice is motivated by the fact that the RGB modality inherently provides richer spatial resolution, fine-grained textures, and structural details compared to the thermal modality. Routing the fused representation through the RGB-side pathway ensures that these critical geometric features are better preserved for the subsequent dense prediction task. Consequently, $E_{k}^{S}$ is applied to the fused representation rather than to an RGB-only feature. The decoder is kept identical to that of EAEFNet, ensuring that the proposed modification is restricted to the encoder branch topology.

### 3.2. Transition-Stage Analysis

The transition stage is governed by two competing requirements. If the RGB and thermal branches are unified before each modality has formed sufficiently informative responses, modality-specific cues such as appearance texture, visible-spectrum boundaries, and thermal contrast may be inadequately preserved. Conversely, if the two modalities progressively encode more aligned task-level structures, maintaining independent transformations through the remaining encoder stages may introduce unnecessary computation. We therefore examine how the relationship between RGB and thermal representations changes with encoder depth in the absence of cross-modal interaction.

To isolate the intrinsic stage-wise relationship from the effect of EAEF operations, we construct independently trained RGB-only and thermal-only reference networks for each dataset–backbone setting. All EAEF operations are removed, and each unimodal network is optimized separately using the same segmentation objective and training protocol. We consider ResNet-18, ResNet-50, and ResNet-152 backbones, indexed by *b*. Because the two reference networks do not exchange features during either training or inference, any measured alignment reflects representation structures learned independently from the two modalities rather than alignment induced by an explicit fusion operation.

For the *i*-th paired RGB–thermal sample, let $A_{i,k}^{m,b}$ denote the output feature map of the independently trained encoder for modality m∈{R,T} at stage *k*, where

$$A_{i,k}^{m,b}\in R^{C_{k}^{b}\times H_{k}^{b}\times W_{k}^{b}}.$$

Each feature map is globally average-pooled to obtain a sample-level descriptor:

(3) $$x_{i,k}^{m,b}=\frac{1}{H_{k}^{b}W_{k}^{b}}\sum_{h=1}^{H_{k}^{b}}\sum_{w=1}^{W_{k}^{b}}A_{i,k}^{m,b}\left(:,h,w\right)\in R^{C_{k}^{b}}.$$

The *N* descriptors are stacked in paired-sample order, and their linear Gram matrix is defined as

(4) $$X_{k}^{m,b}=\left[\begin{array}{c} {\left(x_{1,k}^{m,b}\right)}^{T}\\ \vdots \\ {\left(x_{N,k}^{m,b}\right)}^{T} \end{array}\right],\;K_{k}^{m,b}=X_{k}^{m,b}{\left(X_{k}^{m,b}\right)}^{T}.$$

We use linear centered kernel alignment (CKA) to compare the sample-level representation structures of the two independently optimized encoders [29]. Let $H_{N}=I_{N}-N^{-1}11^{T}$ be the centering matrix and

(5) $${\tilde{K}}_{k}^{m,b}=H_{N}K_{k}^{m,b}H_{N}$$

be the centered Gram matrix. The stage-wise linear CKA is then computed as

(6) $${\mathrm{CKA}}_{k}^{b}=\frac{{\langle {\tilde{K}}_{k}^{R,b},{\tilde{K}}_{k}^{T,b}\rangle }_{F}}{{∥{\tilde{K}}_{k}^{R,b}∥}_{F}{∥{\tilde{K}}_{k}^{T,b}∥}_{F}}.$$

Higher CKA indicates greater similarity between the RGB-only and thermal-only sample-relation structures. Its invariance to orthogonal transformations and isotropic scaling makes it suitable for comparing representations learned by independently optimized encoders.

**Figure 2:**
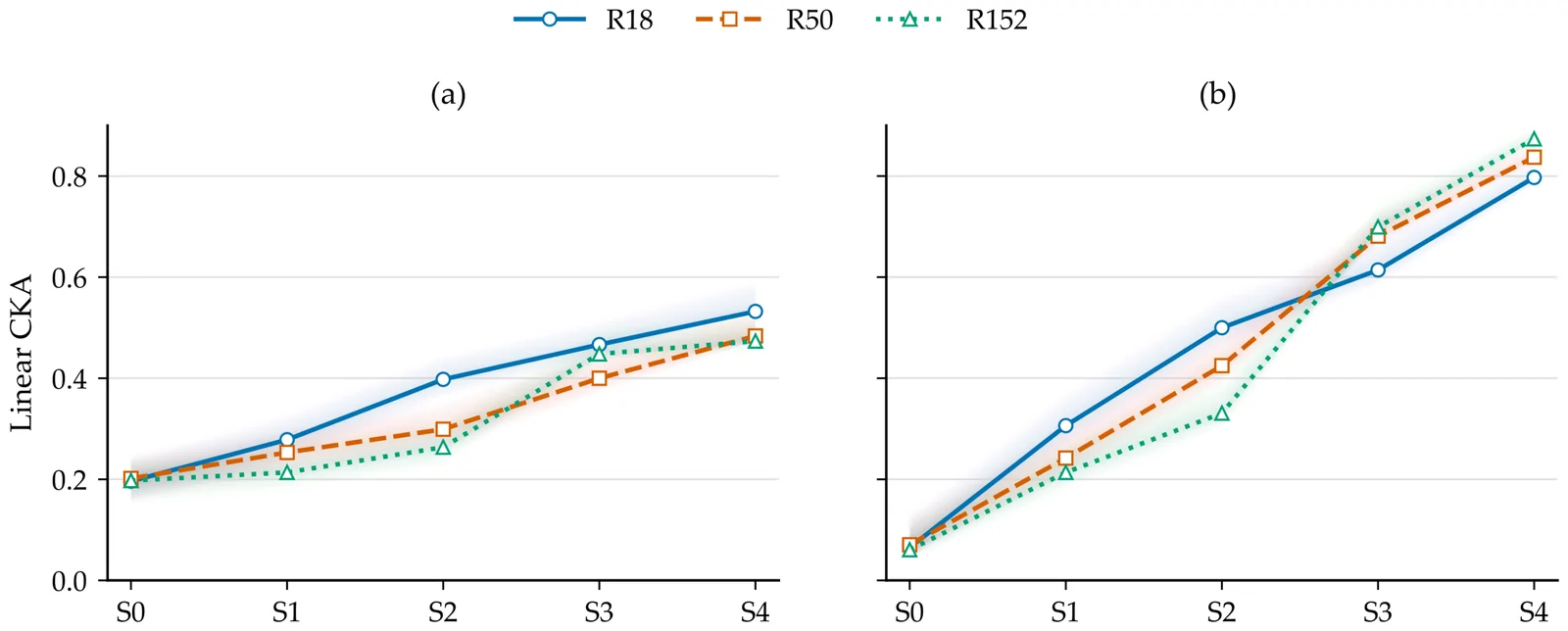
Stage-wise linear CKA between independently trained RGB-only and thermal-only encoders on (**a**) MFNet and (**b**) FMB. For each backbone, all EAEF operations are removed, and the two unimodal networks are optimized separately using the same segmentation protocol. CKA is computed from globally pooled encoder features at s0–s4 using paired RGB–thermal samples. Higher values indicate greater alignment between the independently learned representation structures.

As shown in Figure 2, linear CKA increases from s0 toward s4 for all dataset–backbone settings. The increase is moderate on MFNet and more pronounced on FMB, showing that the degree of alignment remains dataset-dependent. Because the two unimodal networks are trained independently and contain no cross-modal fusion, this stage-wise increase cannot be attributed to feature exchange between the modalities. Instead, it indicates that the common segmentation objective drives the deeper RGB-only and thermal-only encoders toward more similar sample-level representation structures as semantic abstraction increases.

The relatively low CKA at the early stages indicates that the two modalities organize samples differently when encoding modality-dependent appearance and thermal cues. Sharing the encoder too early may therefore restrict the formation of complementary unimodal representations. In contrast, the greater alignment at deeper stages suggests that maintaining two fully separate transformation paths may provide diminishing representational differentiation relative to the associated costs in terms of active parameters and FLOPs. Nevertheless, CKA measures representation geometry rather than task discriminability, cross-modal complementarity, or segmentation accuracy. Furthermore, because our CKA computation relies on globally pooled features, it captures image-level representational alignment but inevitably discards the fine-grained spatial information that is critical for dense prediction tasks. It therefore serves as a qualitative representational motivation for considering the transition stage as an architectural design variable, rather than as a principled selection criterion that identifies the optimal stage for the best accuracy–efficiency trade-off.

Accordingly, we construct transition-stage variants from s0 to s4. The earliest transition, s0, maximally reduces modality-specific computation by routing the fused representation to the single shared branch immediately after the stem-level stage. The intermediate variants, s1–s3, retain separate RGB and thermal processing over progressively larger portions of the shallow-to-middle feature hierarchy. The latest transition, s4, preserves the modality-specific branches until the deepest encoder stage. All variants use the same EAEF operation and decoder and differ only in the transition index τ. The following subsection instantiates the proposed network by comparing candidate backbone–transition configurations and fixing a single configuration for the subsequent experimental validation.

### 3.3. Network Configuration

The preceding representational analysis establishes the transition stage as an architectural design variable, but representational alignment alone does not determine task performance or computational efficiency. We therefore instantiate the proposed network by comparing candidate configurations formed by combining transition stages s0–s4 with ResNet-18, ResNet-50, and ResNet-152 backbones. All transition-stage variants retain the same EAEF operation and decoder. Within each dataset and backbone, the loss function, data augmentation, input resolution, and training schedule are also kept unchanged, such that the comparison isolates the effect of the transition stage. The common implementation and evaluation protocol is described in Section 4.1.

Table 1 summarizes the configuration-level accuracy–efficiency characteristics using active parameters (i.e., trainable parameters executed along the active forward path), FLOPs, FPS, and mean Intersection over Union (mIoU) on MFNet and FMB. To verify the stability and robustness of the proposed architecture against initialization variations, all candidate configurations were evaluated across both datasets using widely used representative seeds (0, 42, and 3407), and their peak performances are reported in the quantitative comparisons. As a representative example, across 3 independent runs on MFNet, the ResNet-50-s1 configuration achieved a mean test mIoU of 57.70% with a sample standard deviation of 0.85%p (individual peak mIoUs of 56.96%, 57.50%, and 58.63%), providing evidence of reasonable stability across initializations. For completeness, the detailed mean and standard deviation results across all independent runs are provided in the Supplementary Materials.

Among all proposed transition-stage variants, ResNet-50-s1 achieves the highest MFNet mIoU of 58.6%. Relative to the native EAEFNet-ResNet-50 reference, it improves mIoU by 2.7 percentage points while reducing active parameters from 77.9 M to 29.1 M and FLOPs from 109.1 G to 78.0 G, corresponding to reductions of 62.6% and 28.5%, respectively. Its throughput also increases from 30.4 FPS to 45.9 FPS, representing a 51.0% improvement.

The two dataset columns further clarify the trade-off between the ResNet-50 configurations. ResNet-50-s1 exceeds s2 by 1.0 percentage point on MFNet, whereas s2 exceeds s1 by 1.0 percentage point on FMB. However, s1 uses 2.2 M fewer active parameters and 5.8 G fewer FLOPs and runs 2.3 FPS faster at the reported efficiency measurement setting. Moreover, s4 gives the lowest mIoU within every backbone group on both datasets, indicating that preserving separate modality-specific branches until the deepest encoder stage does not yield a corresponding accuracy benefit despite retaining the largest active forward path.

Building upon the transition stage configurations in Table 1, we conducted an additional ablation study to validate the single-point fusion strategy. To evaluate the effect of the fusion strategy within the context of the proposed dual-to-single architecture, we compared the proposed single-stage fusion (Single-EAEF) against multi-stage fusion (Multi-EAEF) on MFNet. Single-EAEF fuses features exclusively at the target stage, whereas Multi-EAEF repeats the identical module across prior stages. As shown in Table 2, despite increasing active parameters and computational overhead, Multi-EAEF yields comparable or lower segmentation accuracy. This result suggests that forcing uniform feature interactions at early shallow stages may amplify cross-modal noise and increase optimization difficulty. While this ablation isolates the effect of the fusion strategy rather than the independent contribution of branch removal, it demonstrates that Single-EAEF avoids additional structural redundancy while achieving competitive or superior accuracy and efficiency within our specific topological design.

Considering the optimal transition-stage trade-offs (Table 1) and the structural efficiency of the single-point fusion strategy (Table 2) alongside computational overhead, we adopt the s1 configuration employing the Single-EAEF strategy—denoted as EAEFNet-ResNet-50-s1—as the representative network used throughout the remainder of this study. Accordingly, all subsequent quantitative and qualitative comparisons use EAEFNet-ResNet-50-s1 with both the backbone and transition stage fixed.

## 4. Experimental Results

### 4.1. Datasets, Implementation Details, and Evaluation Metrics

To evaluate the proposed network, we conduct experiments on MFNet and FMB. We follow their official training and test splits and train and evaluate the network separately on each dataset.

All models are implemented in PyTorch 2.5.1 [30] and trained on an NVIDIA GeForce RTX 3090. The proposed transition-stage variants were developed by modifying the official EAEFNet implementation, which is publicly available at https://github.com/FreeformRobotics/EAEFNet (accessed on 16 July 2026). Specifically, we modified the encoder topology to introduce the dual-to-single-encoder transition, while using the original EAEF operation without modification at the selected transition stage and retaining the native decoder architecture. ResNet-18, ResNet-50, and ResNet-152 encoders use ImageNet-pretrained weights [31], and the thermal stem is initialized by averaging the pretrained RGB kernels across the input channels. Training is performed for 100 epochs using stochastic gradient descent (SGD) with a batch size of 3, an initial learning rate of 0.02, a momentum of 0.9, a weight decay of $5\times 10^{-4}$, and a per-epoch decay factor of 0.95. Random horizontal flipping (p=0.5) and random cropping (crop rate 0.1) are applied. The objective sums the two output losses, which are derived from the multi-scale representations within the decoder, each defined as an equally weighted combination of multi-class Dice loss [32] and soft cross-entropy loss.

At inference, the two segmentation logits are summed before the final argmax to fuse the multi-scale predictions, thereby improving the overall prediction stability and segmentation robustness. We report class-wise Intersection over Union (IoU) and its mean (mIoU). For class *i*, let $TP_{i}$, $FP_{i}$, and $FN_{i}$ denote the numbers of true-positive, false-positive, and false-negative pixels, respectively. IoU and mIoU are computed as

(7) $${\mathrm{IoU}}_{i}=\frac{TP_{i}}{TP_{i}+FP_{i}+FN_{i}},$$

(8) $$\mathrm{mIoU}=\frac{1}{\left|C\right|}\sum_{i\in C}{\mathrm{IoU}}_{i},$$

where C denotes the evaluated class set. MFNet mIoU is averaged over all nine classes. For FMB, the *bicycle* class is retained during training but excluded from test mIoU because it is absent from the public test split; mIoU is therefore averaged over the remaining 14 evaluated classes.

FLOPs and FPS are measured at 480×640 for MFNet and 600×800 for FMB, with FPS measured using a batch size of 1 on the same RTX 3090. Specifically, inputs were preloaded to the GPU, and measurements were conducted in FP32 precision. The protocol included 100 warm-up iterations followed by three separate trials of 200 timed inference iterations using CUDA events. CUDA synchronization was strictly enforced after the warm-up phase and during each iteration, and the final throughput is reported as the median FPS across the three trials.

In our implementation for this internal configuration analysis, unused modality-specific branches are simply excluded from the active forward computation, whereas they are physically removed from the deployed model for the state-of-the-art comparison.

### 4.2. Comparison with State-of-the-Art Methods

This subsection evaluates the fixed EAEFNet-ResNet-50-s1 configuration established in Section 3.3 against existing RGB-T semantic segmentation methods. We first use MFNet to analyze overall segmentation accuracy, computational efficiency, and daytime/nighttime robustness, and then present a cross-method comparison on FMB. A qualitative comparison on MFNet relates the quantitative trends to representative prediction maps.

Table 3 presents the overall quantitative comparison on the MFNet test set. EAEFNet-ResNet-50-s1 achieves 58.6% mIoU, matching AGFNet and remaining close to the native EAEFNet-ResNet-152 reference value of 58.9%. Recent methods such as CRM-RGBTSeg (CRM), MiLNet, and CMX-B4 report higher overall mIoU, but the representative configuration maintains competitive segmentation accuracy through a compact active forward path. The class-wise results further show that the strongest categories are distributed across methods rather than concentrated in a single architecture.

Table 4 provides a more direct view of the MFNet accuracy–efficiency comparison. EAEFNet-ResNet-50-s1 records the same 58.6% mIoU as AGFNet while using 29.1 M parameters and 78.0 G FLOPs, compared with the reported 72.2 M parameters and 219.1 G FLOPs of AGFNet. The corresponding reported throughput values are 45.9 FPS and 15.3 FPS, respectively. ECFNet reports a throughput of 55.6 FPS with 56.2% mIoU, while RTNet reports 48.0 M parameters, 46.8 G FLOPs, 40.2 FPS, and 58.0% mIoU. Because the cross-method FPS values are obtained under method-specific measurement environments, they are interpreted as indicative reported values rather than as a controlled hardware comparison. Taken together, the results place EAEFNet-ResNet-50-s1 in a favorable region where competitive mIoU, moderate FLOPs, and high reported throughput are jointly maintained. This trend is consistent with the configuration analysis, which shows that transition-stage design can reduce redundant modality-specific computation without forcing a large accuracy drop.

Table 5 reports the daytime and nighttime comparison on MFNet. EAEFNet-ResNet-50-s1 obtains 49.7% mIoU in daytime scenes and 58.9% mIoU in nighttime scenes. Although SpiderMesh, CMX-B2, CMX-B4, and NLFNet report higher daytime scores, the nighttime score of EAEFNet-ResNet-50-s1 remains close to the strongest reported value, 59.4% from CMX-B4. This behavior is consistent with the role of thermal information in RGB-T perception under low illumination. It indicates that, even after the modality-specific RGB and thermal branches are unified into a single shared branch at the selected transition stage, the representative configuration is consistent with the retention of useful multimodal cues for nighttime segmentation, although these results do not explicitly isolate the continued active exploitation of thermal features within the shared branch.

**Figure 3:**
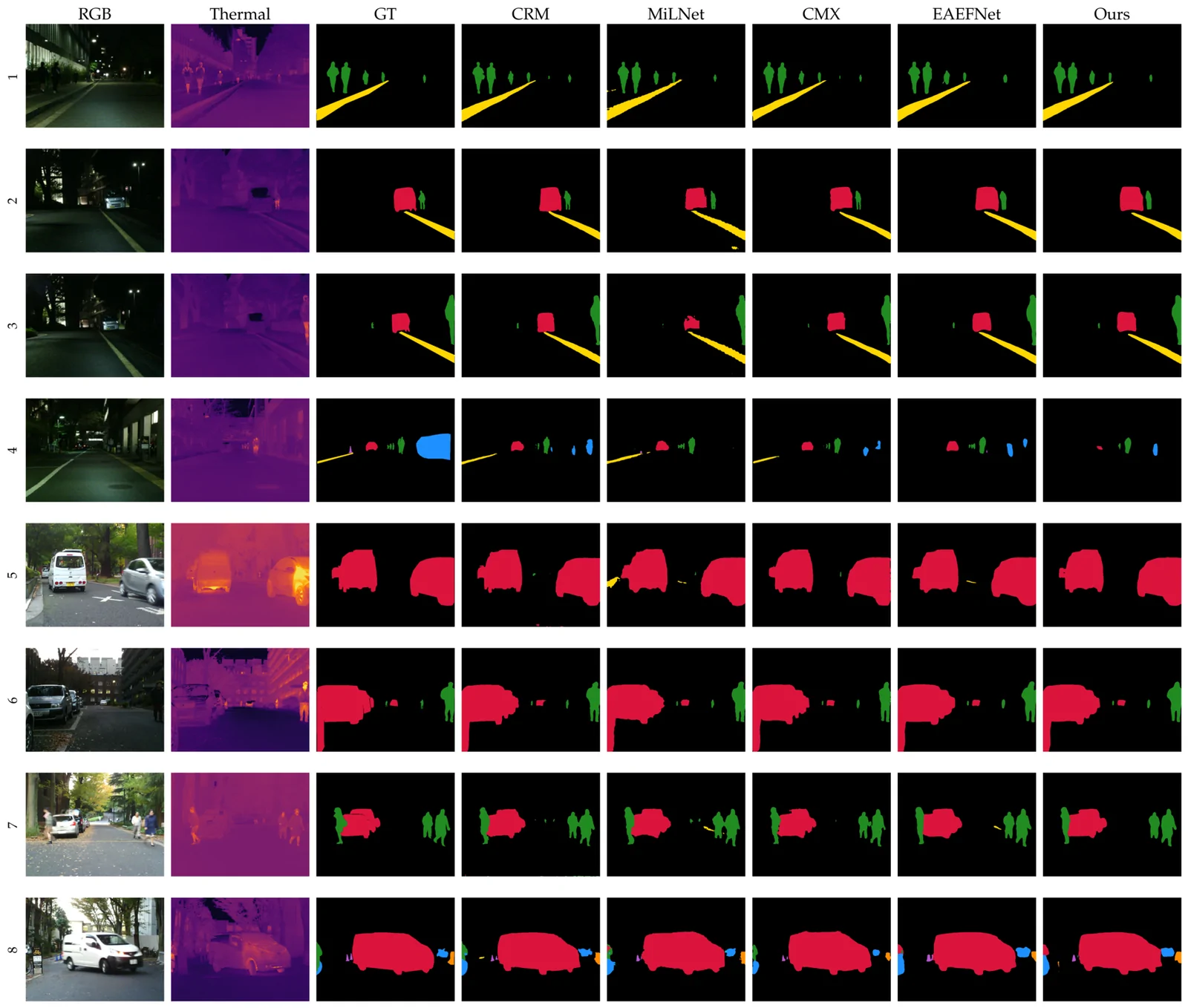
Qualitative comparison on MFNet. The numbers 1–8 denote the indices of the selected test samples. Each row shows the RGB image, thermal image, ground truth, and segmentation outputs of CRM, MiLNet, CMX, native EAEFNet, and EAEFNet-ResNet-50-s1 (ours), including both nighttime and daytime examples. The colors in the segmentation masks represent different semantic classes (e.g., red for car, green for person, blue for bike, and yellow for curve).

Figure 3 shows qualitative MFNet examples for RGB images, thermal images, ground-truth masks, and the segmentation outputs of CRM, MiLNet, CMX, native EAEFNet, and EAEFNet-ResNet-50-s1. To provide a transparent evaluation, rows 1–3 and 5–7 display successful predictions with high mIoU, while rows 4 and 8 illustrate representative failure cases.

In the successful low-illumination and daytime examples, EAEFNet-ResNet-50-s1 effectively preserves dominant scene layouts, compact person and vehicle regions, and main road–curve structures. This qualitative behavior aligns with the competitive nighttime mIoU in Table 5, indicating that the performance trend is consistent with the retention of useful multimodal information.

However, as observed in the failure cases (rows 4 and 8), our model occasionally struggles with highly cluttered or ambiguous scenes, resulting in fragmented or omitted predictions for small-scale or overlapping objects. We attribute these limitations to the inherent trade-off of our single-stage fusion strategy. Because multimodal integration is restricted to a single transition stage to maximize real-time efficiency, any subtle complementary cues that fail to be effectively fused at this specific bottleneck cannot be recovered in subsequent layers. Nevertheless, the proposed configuration maintains coherent object-level predictions overall, visually supporting the table-level finding that its strength lies in a balanced accuracy–efficiency profile rather than in maximizing every class-wise score.

After establishing the MFNet results, Table 6 presents the cross-method comparison on FMB. EAEFNet-ResNet-50-s1 achieves 60.5% mIoU, matching AGFNet and exceeding RTNet, SegMiF, and ECFNet in overall score. The class-wise results reveal complementary strengths across methods: RTNet performs strongly on Car, Building, and Vegetation; AGFNet obtains the highest Pole IoU; SegMiF gives the highest Truck IoU; and EAEFNet-ResNet-50-s1 achieves the highest Person, T-Lamp, and T-Sign scores. This distribution shows that the strongest class-wise method varies by semantic category, while the representative configuration maintains competitive overall mIoU in the cross-method comparison. For completeness, the detailed class-wise IoU results for all 14 evaluated FMB categories are provided in the Supplementary Materials.

Overall, the comparative results show that the proposed dual-to-single-encoder transition strategy provides a meaningful accuracy–efficiency balance beyond the controlled configuration analysis. On MFNet, EAEFNet-ResNet-50-s1 achieves competitive mIoU with a compact active forward path, while the daytime/nighttime comparison and qualitative examples indicate that useful multimodal cues are retained after the transition. The cross-method FMB comparison further shows that the same representative configuration remains competitive against existing RGB-T segmentation methods. Together, these results support the main claim that transition-stage design is an effective way to improve the practical accuracy–efficiency trade-off in RGB-T semantic segmentation.

## 5. Conclusions and Discussion

### 5.1. Conclusions

We proposed a dual-to-single-encoder transition strategy for real-time RGB-T semantic segmentation to explore its potential applicability in resource-constrained environments. To reduce the computational burden of conventional dual-branch encoders, the proposed strategy preserves modality-specific RGB and thermal branches only up to a selected transition stage, applies the corresponding EAEF operation, and routes the fused representation through a single shared branch for the remaining encoder stages. By treating the transition stage as an architectural design variable, the proposed strategy provides a practical alternative to maintaining fully active dual-branch, multi-level fusion topologies throughout the entire encoder. The experimental results show that early-to-middle transitions offer a favorable accuracy–efficiency trade-off. In particular, on MFNet, EAEFNet-ResNet-50-s1 reduces parameters and FLOPs by approximately 62.6% and 28.5%, respectively, relative to the native EAEFNet-ResNet-50 reference, while improving mIoU by 2.7 percentage points and increasing inference throughput from 30.4 FPS to 45.9 FPS. Overall, this study demonstrates that, within the evaluated EAEFNet-based architectures and ResNet backbones, effective RGB-T fusion does not require modality-specific RGB and thermal branches to remain active until the final encoder stage. Accordingly, selecting an appropriate transition stage can preserve useful cross-modal representations while reducing redundant modality-specific computation, thereby highlighting its potential applicability for future resource-constrained deployments.

### 5.2. Limitations and Future Work

While the proposed framework effectively reduces the computational overhead of RGB-T semantic segmentation, several limitations remain. First, the framework’s behavior and the selected transition stages are primarily validated on an EAEFNet-based design; generalizing these findings to other fusion architectures requires further investigation. Second, the cross-method comparisons involve heterogeneous runtime conditions, and the potential for run-to-run variability necessitates cautious interpretation of the absolute performance metrics. Third, our current CKA analysis utilizes globally pooled descriptors, which inherently discard fine-grained spatial information. Finally, although we target real-time edge applications, this study lacks direct validation on embedded devices. In future work, we plan to address these limitations by exploring dynamic, input-adaptive transition strategies; investigating stage-adaptive hierarchical fusion mechanisms to mitigate the structural bottlenecks of uniform multi-stage fusion; and extending our representational analysis to retain spatial dimensions. Furthermore, we aim to implement and co-optimize the proposed shared-branch architecture on dedicated hardware accelerators, such as FPGAs, to explicitly evaluate and maximize latency and power efficiency in real-world edge environments.

## Figures and Tables

**Table 1:** Comparison of the proposed EAEFNet transition-stage configurations. Active parameters denote the trainable parameters executed along the active forward path. FLOPs and FPS are measured at the MFNet input resolution of 480×640, with FPS measured on an NVIDIA GeForce RTX 3090 using a batch size of 1. The mIoU values are reported on MFNet and FMB under their respective dataset-specific protocols. Boldface denotes the network configuration adopted throughout the subsequent experiments; native EAEFNet rows are included as references where results are available. To ensure direct comparability with the native baseline and establish consistency with the standardized evaluation protocol in RGB-T semantic segmentation, the mIoU values for all configurations are evaluated on the official test splits.

Backbone	Stage	Active Params (M)	FLOPs (G)	FPS		mIoU (%)	
				RTX 3090		MFNet	FMB
ResNet-152	s0	63.2	118.7	36.2		57.2	64.8
	s1	63.7	123.6	33.8		58.1	62.3
	s2	67.0	134.8	31.7		57.8	63.5
	s3	111.3	183.8	26.2		57.7	61.5
	s4	140.6	188.6	25.2		56.1	60.1
	native	147.3	200.4	20.7		58.9	–
ResNet-50	s0	28.6	73.1	50.0		57.0	59.0
	**s1** ^†^	**29.1**	**78.0**	**45.9**		**58.6**	**60.5**
	s2	31.3	83.8	43.6		57.6	61.5
	s3	42.0	92.6	42.6		56.0	59.1
	s4	71.3	97.5	41.2		54.1	57.8
	native	77.9	109.1	30.4		55.9	–
ResNet-18	s0	15.2	56.3	56.4		55.1	57.6
	s1	15.4	59.1	55.3		55.0	58.7
	s2	15.9	61.5	54.7		56.3	58.9
	s3	18.3	64.0	53.1		55.9	59.8
	s4	27.6	66.5	52.4		52.7	56.4

^†^ Denotes the representative network configuration adopted for all subsequent comparisons.

**Table 2:** Comparison of Single-EAEF and Multi-EAEF on the ResNet-50 backbone to evaluate the effect of fusion strategy. Note that Multi-EAEF at stage s4 is structurally equivalent to the native EAEFNet baseline. The evaluation metrics and experimental environment are identical to those detailed in Table 1.

Backbone	Stage	Active Params (M)	FLOPs (G)	FPS		mIoU (%)
				RTX 3090		MFNet
ResNet-50 (Multi-EAEF)	s0–s1	29.1	78.2	43.5		57.0
	s0–s2	31.6	85.1	38.3		57.5
	s0–s3	43.7	94.3	34.7		56.2
	s0–s4 (native)	77.9	109.1	30.4		55.9
ResNet-50 (Single-EAEF)	**s1** ^†^	**29.1**	**78.0**	**45.9**		**58.6**
	s2	31.3	83.8	43.6		57.6
	s3	42.0	92.6	42.6		56.0
	s4	71.3	97.5	41.2		54.1

^†^ Denotes the representative network configuration adopted for all subsequent comparisons.

**Table 3:** Quantitative comparison with existing RGB-T semantic segmentation methods on MFNet, reporting IoU (%) for eight displayed classes and mIoU (%) over all nine classes. Best values in the displayed IoU and mIoU columns, including ties, are shown in bold; ours denotes EAEFNet-ResNet-50-s1.

Method	Backbone	Params (M)	FLOPs (G)	Car	Person	Bike	Curve	Car Stop	Guardrail	Color Cone	Bump	mIoU
MFNet [17]	DCNN	0.7	8.4	65.9	58.9	42.9	29.9	9.9	0.0	25.2	27.7	39.7
ACNet [33]	ResNet-50	116.6	123.8	79.4	64.7	52.7	32.9	28.4	0.8	16.9	44.4	46.3
RTFNet [13]	ResNet-152	245.5	337.1	87.4	70.3	62.7	45.3	29.8	0.0	29.1	55.7	53.2
PSTNet [23]	ResNet-18	20.4	129.4	76.8	52.6	55.3	29.6	25.1	15.1	39.4	45.0	48.4
HRNet [34]	DCNN	132.7	222.6	86.9	67.3	59.2	35.3	23.1	1.7	46.6	47.3	51.7
FuseSeg [35]	DenseNet-161	141.5	193.4	87.9	71.7	64.6	44.8	22.7	6.4	46.9	47.9	54.5
ABMDRNet [36]	ResNet-50	64.6	194.3	84.8	69.6	60.3	45.1	33.1	5.1	47.4	50.0	54.8
FEANet [14]	ResNet-152	-	-	87.8	71.1	61.1	46.5	22.1	6.6	55.3	48.9	55.3
NLFNet [37]	ResNet-18	-	-	88.5	69.0	63.9	47.8	25.6	6.1	45.0	44.7	54.3
GCNet [38]	ResNet-50	49.9	59.3	86.0	72.0	60.0	42.8	30.7	6.2	49.5	52.6	55.3
MMNet [39]	ResNet-18	23.9	95.0	83.9	69.3	59.0	43.2	24.7	4.6	42.2	50.7	52.8
CCFFNet [40]	ResNet-101	109.1	527.5	89.6	74.2	63.1	50.5	31.9	4.8	49.7	56.3	57.6
LASNet [24]	ResNet-152	93.6	234.4	84.2	67.1	56.9	41.1	39.6	**18.9**	48.8	40.1	54.9
MLFNet [41]	ResNet-34	-	-	82.3	68.1	67.3	27.3	30.4	15.7	55.6	40.1	53.8
CMX [15]	MiT-B2	-	-	89.4	74.8	64.7	47.3	30.1	8.1	52.4	59.4	58.2
CMX [15]	MiT-B4	-	-	**90.1**	75.2	64.5	50.2	35.3	8.5	54.2	60.6	59.7
EAEFNet [28]	ResNet-152	147.3	200.4	87.6	72.6	68.3	48.6	35.0	14.2	52.4	58.3	58.9
SGFNet [26]	ResNet-50	125.3	143.7	88.4	**77.6**	64.3	45.8	31.0	6.0	**57.1**	55.0	57.6
MMSMCNet [42]	SegFormer-B3	98.6	181.8	89.2	69.1	63.5	46.4	41.9	8.8	48.8	57.6	58.1
SpiderMesh [43]	MiT-B4	-	398.8	89.9	75.3	64.8	51.5	31.4	4.5	54.5	55.9	58.4
IGFNet [44]	MiT-B2	67.4	69.2	88.0	74.0	62.7	48.2	36.0	14.2	52.4	57.5	59.0
CAINet [45]	MobileNet-V2	12.2	123.6	88.5	66.3	**68.7**	**55.4**	31.5	9.0	48.9	**60.7**	58.6
CRM-RGBTSeg [46]	Swin-B	-	-	90.0	75.1	67.0	45.2	**49.7**	18.4	54.2	54.4	**61.4**
MDBFNet [47]	ResNet-152	97.9	249.3	89.1	73.5	62.6	48.8	34.6	8.0	54.5	51.3	57.8
AGFNet [48]	ResNet-50	72.2	219.1	88.0	73.7	64.6	45.3	34.3	13.8	57.0	52.5	58.6
SCRNet [49]	ResNet-50	294.5	304.1	87.9	74.1	62.7	47.4	36.2	7.5	53.2	54.4	58.0
MiLNet [50]	SegFormer-B3	92.3	136.3	87.9	72.3	64.5	46.2	45.8	10.1	55.8	60.1	60.1
RTNet [51]	DCNN + Transformer	48.0	46.8	88.5	73.9	62.0	48.7	32.2	7.9	52.4	58.0	58.0
ours	ResNet-50	29.1	78.0	86.3	71.6	63.1	49.7	41.1	11.4	51.4	55.0	58.6

**Table 4:** Reported accuracy–efficiency comparison with existing RGB-T semantic segmentation methods on MFNet. The lowest parameter and FLOP counts and the highest FPS and mIoU values, including ties, are shown in bold; ours denotes EAEFNet-ResNet-50-s1. Note that the FPS values for existing methods are reported in the literature and represent indicative throughputs under heterogeneous measurement environments.

Method	Params (M)	FLOPs (G)	FPS	mIoU
LASNet [24]	93.6	234.4	26.8	54.9
SFAF-MA [52]	156.0	354.4	11.2	55.5
SegMiF [18]	45.6	359.4	2.4	56.1
MDRNet [53]	64.6	194.3	20.9	56.8
ECFNet [54]	**21.3**	**40.2**	**55.6**	56.2
SCRNet [49]	294.5	304.1	18.6	58.0
AGFNet [48]	72.2	219.1	15.3	**58.6**
RTNet [51]	48.0	46.8	40.2	58.0
ours	29.1	78.0	45.9	**58.6**

**Table 5:** Segmentation results for daytime and nighttime images from MFNet. The highest mIoU value in each split is shown in bold; ours denotes EAEFNet-ResNet-50-s1.

Method	Backbone	mIoU (%)	
		Daytime	Nighttime
RTFNet [13]	ResNet-152	45.8	54.8
FuseSeg [35]	DenseNet-161	47.8	54.6
NLFNet [37]	ResNet-18	50.3	54.8
SpiderMesh [43]	ResNet-152	52.0	56.0
CMX [15]	MiT-B2	51.3	57.8
CMX [15]	MiT-B4	**52.5**	**59.4**
AGFNet [48]	ResNet-50	49.9	58.7
ours	ResNet-50	49.7	58.9

**Table 6:** Quantitative comparison with existing RGB-T semantic segmentation methods on FMB, reporting IoU (%) for eight displayed classes and mIoU (%) over the 14 evaluated classes. Best values in the displayed IoU and mIoU columns, including ties, are shown in bold; ours denotes EAEFNet-ResNet-50-s1.

Method	Backbone	Car	Person	Truck	T-Lamp	T-Sign	Building	Vegetation	Pole	mIoU
LASNet [24]	ResNet-152	72.6	48.6	14.8	2.9	59.0	75.4	81.6	36.7	42.5
SFAF-MA [52]	ResNet-50	73.0	55.7	14.3	13.6	54.2	73.0	78.9	38.1	42.7
SegMiF [18]	MiT-B3	78.3	65.4	**47.3**	43.1	74.8	82.0	85.0	49.8	54.8
ECFNet [54]	ResNet-18	80.0	63.1	12.8	40.6	71.9	81.4	84.4	44.6	52.5
EGFNet [55]	ResNet-152	77.4	63.0	17.1	25.2	66.6	77.2	83.5	41.5	47.3
AGFNet [48]	ResNet-50	78.9	62.1	36.9	46.3	72.3	81.0	85.4	**63.0**	**60.5**
RTNet [51]	DCNN + Transformer	**83.0**	67.9	35.0	39.5	74.0	**84.5**	**86.8**	51.8	59.1
ours	ResNet-50	82.4	**70.2**	29.7	**46.9**	**75.8**	82.7	86.4	47.6	**60.5**

## Data Availability

The MFNet dataset can be found at https://www.mi.t.u-tokyo.ac.jp/static/projects/mil_multispectral/, accessed on 16 July 2026; the FMB dataset can be found at https://github.com/JinyuanLiu-CV/SegMiF, accessed on 16 July 2026. Both datasets are publicly available, and no new datasets were created in this study.

## Supplementary Materials

The following supporting information can be downloaded at https://www.mdpi.com/article/10.3390/s26206602/s1. Section S1: Statistical Robustness across Independent Runs; Section S2: Detailed Class-wise Results on FMB Dataset.
