# Supplementary material for: Exploring Dual-to-Single-Encoder Transitions for Real-Time RGB-T Semantic Segmentation
Source: Sensors (Basel). 2026 Oct 18;26(20):6602. doi: 10.3390/s26206602 (PMC13709217; doi:10.3390/s26206602)
Supplement: Supplementary file 1 [file sensors-26-06602-s001.zip › sensors-4604921-supplementary.pdf]

Supplementary Materials for

"Exploring Dual-to-Single Encoder Transitions for Real-Time RGB-T Semantic Segmentation"

This supplementary document provides detailed quantitative evaluations to support the main manuscript. Section S1 presents the statistical robustness of the proposed EAEFNet configurations across independent runs for both MFNet and FMB datasets. Section S2 provides the complete class-wise Intersection over Union (IoU) results for all 14 evaluated categories on the FMB dataset, supplementing the abbreviated state-of-the-art comparison in the main text.

S1. Statistical Robustness across Independent Runs

Table S1: Quantitative evaluation of the proposed EAEFNet configurations across different back-bones and fusion sites on MFNet, reporting mean IoU (%) for eight displayed classes and mIoU (%) over all nine classes. The mIoU values represent the mean and standard deviation across three independent runs. Best mean values in the displayed IoU and mIoU columns within each backbone group are shown in bold. The reported parameter count denotes active parameters.

MFNet													
Method	Backbone	Params. (M)	FLOPs (G)	Fusion	Car	Person	Bike	Curve	Car Stop	Guardrail	Color Cone	Bump	mIoU
EAEFNet	R152	63.2	118.7	s0	86.2	70.4	62.3	44.8	34.1	8.5	54.2	56.4	57.0 ± 0.2
		63.7	123.6	s1	87.3	71.5	64.5	48.8	33.4	**12.3**	53.2	53.8	**57.7 ± 0.5**
		67.0	134.8	s2	**87.5**	71.6	**64.9**	**49.8**	**34.4**	9.2	53.7	50.9	57.6 ± 0.3
		111.3	183.8	s3	**87.5**	**71.8**	64.2	47.6	30.7	2.9	**54.6**	**61.6**	56.6 ± 1.4
		140.6	188.6	s4	85.3	66.4	60.7	41.5	32.8	9.3	53.8	57.1	54.8 ± 1.1
		147.3	200.4	native	87.6	72.6	63.8	48.6	35.0	14.2	52.4	58.3	58.9
	R50	28.6	73.1	s0	84.9	**72.4**	61.0	47.6	35.5	**12.6**	45.4	56.0	56.5 ± 0.5
		29.1	78.0	s1	86.3	71.6	**63.1**	**49.7**	**41.1**	11.4	**51.4**	55.0	**57.7 ± 0.9**
		31.3	83.8	s2	**86.5**	72.1	62.2	47.1	37.0	12.4	50.6	52.7	57.3 ± 0.6
		42.0	92.6	s3	85.9	68.2	57.8	47.3	35.6	8.4	44.3	**58.8**	55.7 ± 0.4
		71.3	97.5	s4	84.9	64.7	60.3	38.3	31.3	11.7	50.8	47.4	53.5 ± 0.5
		77.9	109.1	native	86.8	71.8	62.0	49.7	29.7	7.1	50.9	46.7	55.9
	R18	15.2	56.3	s0	83.5	67.8	60.6	**47.5**	33.0	6.3	44.4	**54.9**	54.4 ± 1.0
		15.4	59.1	s1	85.2	70.2	62.0	47.1	29.6	5.7	49.1	48.1	54.6 ± 0.7
		15.9	61.5	s2	**86.3**	**71.4**	60.1	43.6	31.9	7.8	**54.6**	52.9	**55.7 ± 0.7**
		18.3	64.0	s3	83.7	69.2	**62.6**	44.6	**35.3**	**8.5**	48.7	52.6	55.1 ± 0.8
		27.6	66.5	s4	83.4	65.2	59.7	41.2	32.0	1.9	44.3	48.9	52.3 ± 0.6
		–	–	native	–	–	–	–	–	–	–	–	–

Table S2: Quantitative evaluation of the proposed EAEFNet configurations across different back-bones and fusion sites on FMB, reporting mean IoU (%) for eight displayed classes and mIoU (%) over the 14 evaluated classes. The mIoU values represent the mean and standard devia-tion across three independent runs. Best mean values in the displayed IoU and mIoU columns within each backbone group are shown in bold. The reported parameter count denotes active parameters.

FMB													
Method	Backbone	Params. (M)	FLOPs (G)	Fusion	Car	Person	Truck	T-Lamp	T-Sign	Building	Vegetation	Pole	mIoU
EAEFNet	R152	63.2	188.2	s0	**83.2**	**71.3**	**43.1**	**51.2**	**77.5**	83.6	86.7	49.1	**62.3 ± 2.3**
		63.7	195.9	s1	82.9	**71.3**	29.2	47.4	75.5	84.1	86.7	48.6	61.9 ± 0.4
		67.0	213.6	s2	82.7	70.9	39.4	45.9	76.7	**84.6**	**87.2**	**49.3**	62.1 ± 1.2
		111.3	291.2	s3	**83.2**	68.6	41.7	48.5	75.1	82.6	86.3	48.3	60.8 ± 0.6
		140.6	298.9	s4	80.6	60.6	29.3	47.5	76.1	81.6	85.7	46.6	59.8 ± 0.3
	R50	28.6	116.0	s0	80.6	69.8	18.5	40.9	75.8	82.7	**86.4**	47.1	58.8 ± 0.3
		29.1	123.7	s1	**82.4**	**70.2**	29.7	46.9	75.8	82.7	**86.4**	**47.6**	60.2 ± 0.3
		31.3	132.9	s2	81.5	69.4	**43.2**	**47.6**	**76.8**	**83.8**	**86.4**	46.8	**61.0 ± 0.6**
		42.0	146.8	s3	80.7	65.6	36.9	43.2	75.5	82.1	85.3	**47.6**	58.7 ± 0.4
		71.3	154.5	s4	80.9	59.2	36.4	43.2	75.2	80.3	84.8	45.5	57.7 ± 0.3
	R18	15.2	89.3	s0	79.7	67.3	29.2	34.6	71.9	81.9	**86.0**	44.3	57.4 ± 0.2
		15.4	93.8	s1	79.6	**68.8**	29.2	40.4	**76.0**	82.3	85.9	46.5	58.2 ± 0.7
		15.9	97.6	s2	**81.3**	67.7	37.7	36.3	74.7	**83.1**	**86.0**	**46.7**	**58.4 ± 0.5**
		18.3	101.5	s3	80.1	66.3	**40.6**	**44.5**	75.7	82.1	85.9	45.4	**58.4 ± 1.4**
		27.6	105.5	s4	79.3	57.9	32.1	39.2	73.9	81.5	84.2	43.9	55.8 ± 0.8

S2. Detailed Class-wise Results on FMB Dataset

Table S3: Quantitative comparison with existing RGB-T semantic segmentation methods on FMB, reporting class-wise IoU (%) and mIoU (%) over all 14 evaluated classes. Best values in each column are shown in bold.

**FMB Network Comparison (14 Classes)**																
Method	Backbone	Car	Person	Truck	T-Lamp	T-Sign	Building	Vegetation	Pole	Background	Road	Sidewalk	Sky	Bus	Motorcycle	mIoU
LASNet [24]	ResNet-152	72.6	48.6	14.8	2.9	59.0	75.4	81.6	36.7	–	–	–	–	–	–	42.5
SFAF-MA [52]	ResNet-50	73.0	55.7	14.3	13.6	54.2	73.0	78.9	38.1	–	–	–	–	–	–	42.7
SegMiF [18]	MiT-B3	78.3	65.4	**47.3**	43.1	74.8	82.0	85.0	49.8	–	–	–	–	–	–	54.8
ECFNet [54]	ResNet-18	80.0	63.1	12.8	40.6	71.9	81.4	84.4	44.6	–	–	–	–	–	–	52.5
EGFNet [55]	ResNet-152	77.4	63.0	17.1	25.2	66.6	77.2	83.5	41.5	–	–	–	–	–	–	47.3
AGFNet [48]	ResNet-50	78.9	62.1	36.9	46.3	72.3	81.0	85.4	**63.0**	–	–	–	–	–	–	**60.5**
RTNet [51]	DCNN+Transformer	**83.0**	67.9	35.0	39.5	74.0	**84.5**	**86.8**	51.8	–	–	–	–	–	–	59.1
ours	EAEFNet-ResNet-50-s1	82.4	**70.2**	29.7	**46.9**	**75.8**	82.7	86.4	47.6	18.1	89.6	52.9	94.5	31.4	39.1	**60.5**
